# Blockade efficacy of MEK/ERK-dependent autophagy enhances PI3K/Akt inhibitor NVP-BKM120's therapeutic effectiveness in lung cancer cells

**DOI:** 10.18632/oncotarget.11645

**Published:** 2016-08-27

**Authors:** Hui Ren, Hua Guo, Asmitananda Thakur, Shuo Zhang, Ting Wang, Yiqian Liang, Puyu Shi, Lei Gao, Feng Liu, Jing Feng, Tianjun Chen, Tian Yang, Dong Shang, Johnson J. Liu, Feng Xu, Mingwei Chen

**Affiliations:** ^1^ Department of Respiratory and Critical Care Medicine, The First Affiliated Hospital of Xi'an Jiaotong University, Xi'an, Shaanxi, China; ^2^ Bioinspired Engineering and Biomechanics Center, Xi'an Jiaotong University, Xi'an, Shaanxi, China; ^3^ Department of Respiratory Medicine, Xi'an Central Hospital, Xi'an, Shaanxi, China; ^4^ Department of Internal Medicine, Life Guard Hospital, Biratnagar, Nepal; ^5^ Department of Pharmacology, School of Medical Sciences, University of New South Wales, Sydney, Australia

**Keywords:** autophagy, apoptosis, BKM120, ERK, lung cancer

## Abstract

NVP-BKM120 (BKM120) is a new pan-class I phosphatidylinositol-3 kinase (PI3K) inhibitor and has been tested in clinical trials as an anticancer agent. In this study, we determined whether BKM120 induces autophagy and the impact of autophagy induction on BKM120's growth-inhibitory activity. BKM120 potently induced elevation of autophagosome-bound type II LC3 (LC3-II) protein, predominantly in cell lines insensitive to BKM120, thereby inducing autophagy. The presence of lysosomal protease inhibitor chloroquine further enhanced the levels of LC3-II. BKM120 combined with chloroquine, enhanced growth-inhibitory effects including induction of apoptosis, suggesting that autophagy is a protective mechanism counteracting BKM120's growth-inhibitory activity. Interestingly, BKM120 increased p-ERK1/2 levels. When blocking the activation of this signaling with MEK inhibitors or with knockdown of ERK1/2, the ability of BKM120 to increase LC3-II was attenuated and the growth-inhibitory effects including induction of apoptosis were accordingly enhanced, suggesting that the MEK/ERK activation contributes to BKM120-induced authophagy. In mouse xenograft model, we also found that the combination of BKM120 and PD0325901 synergistically suppressed cell growth in human lung cancer cells. Thus, the current study not only reveals mechanisms accounting for BKM120-induced autophagy, but also suggests an alternative method to enhance BKM120's therapeutic efficacy against non-small cell lung cancer(NSCLC) by blocking autophagy with either a lysosomal protease inhibitor or MEK inhibitor.

## INTRODUCTION

By far, lung cancer is still the most deadly cancer-related disease [1]. Due to late diagnosis and limited efficacy of available pharmacological treatments, survival rates for lung cancer have not significantly improved in the last several decades. Personalized pharmacotherapy is gaining popularity in clinical oncology. Human genome sequencing has been legalized which will bring more potential therapeutic targets [2–4]. Therefore, understanding the molecular mechanisms and developing novel therapeutic targets for lung cancer are imperative.

Autophagy plays a key role in degrading redundant or dysfunctional cellular organelles and proteins in the living cells [5]. To our best knowledge, mammalian target of rapamycin (mTOR) which forms a complex with raptor (i.e., mTOR complex 1, mTORC1), is one of the key regulators of autophagy. It plays a vital role in shutting off autophagy with the different conditions of growth factors and nutrients [5, 6]. Accordingly, inhibition of the class I phosphatidylinositol-3 kinase (PI3K)/Akt/mTORC1 axis (e.g., mTOR inhibitor rapamycin) induces autophagy [5]. In different circumstances, killing or protecting cells by autophagy autophagy can be either a death or pro-survival mechanism [5, 6], which may direct personalized cancer therapy.

NVP-BKM120 (BKM120) is a highly selective pan-class I PI3K inhibitor in phase I clinical trials. Previous preclinical studies have shown that BKM120 at tolerated doses can suppress proliferation and induce apoptosis in NSCLC cells, as well as inhibit tumors in mouse xenograft [7–10]. One group reported the synergistic effect of BKM120 and dexamethasone combination, in dexamethasone-sensitive multiple myeloma cells [10]. Moreover, it was recently shown that BKM120 impairs BRCA1/2 expression and sensitizes BRCA proficient triple negative breast cancer to PARP inhibition [11]. Our previous study has shown that, when combined with the mTOR inhibitor RAD001, it synergistically inhibits cell growth [9]. A recent phase I clinical trial, demonstrates that BKM120 exerts target inhibition and preliminary tumor suppressing activity and is safe at the maximum-tolerated dose with clear pharmacokinetic evidence. [12].

Our recent study has demonstrated that BKM120 effectively suppresses cell growth in human lung carcinoma cells accompanied with potent inhibition on the PI3K/Akt/mTOR signaling [9]. In this study, we want to figure out whether BKM120 induces autophagy. Besides that, we also want to understand the impact of autophagy induction on BKM120's growth-inhibitory activity. We also explore the potential mechanisms accounting for BKM120-induced autophagy.

## RESULTS

### BKM120 induces autophagy in human NSCLC cells

**Figure 1:**
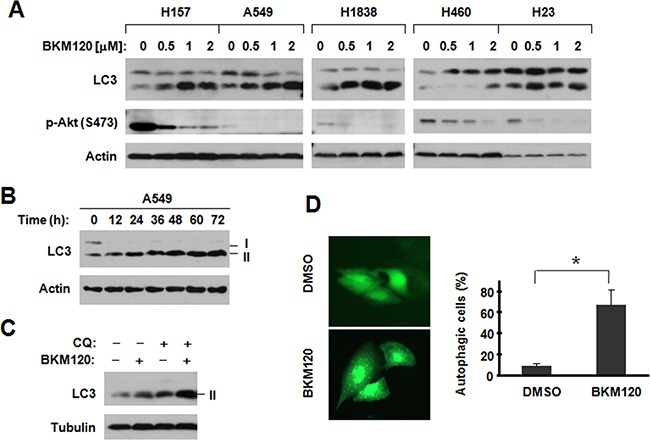
BKM120 induces autophagy in human NSCLC cells **A.** The given cell lines were treated with different concentrations of BKM120 as indicated for 30 h; **B.** A549 cells were treated with 2 μM BKM120 for the indicated times; **C.** A549 cells were treated with 1 μM BKM120, 20 μM CQ or their combination for 30 h. After the aforementioned treatments, the cells were harvested for preparation of whole-cell protein lysates and subsequent Western blot analysis to detect the indicated proteins. **D.** A549/YFP-LC3 cells were treated with 2 μM BKM120 for 48 h and the cells with punctate YFP-LC3 patterns were counted (300 cells/treatment) and pictured. The data are means ± SDs of duplicate determinations. * *P*<0.05.

When treating cells with BKM120 ranging from 0.5 to 2 μM, the expression of LC3-II, were detected increased in cells which effectively inhibited Akt phosphorylation by Western blotting, (Figure 1A). In our previous data, we divided tested cell lines to two groups by IC_50_ of BKM120. We noted that stronger induction of LC3-II was observed in H157, A549 and H1838 cells, which are relatively insensitive to BKM120, than in H460 and H23 cell lines, which are relatively sensitive to BKM120 [9]. Increased levels of LC3-II were detected in cells exposed to BKM120 for 12 h and up to 72 h (Figure 1B). Since LC3-II itself is degraded by autophagy and its increase can be caused due to the accumulation of the autophagosome by blockage of autophagic degradation [13], we further determined whether BKM120 indeed induces autophagic flux. In order to understand the modulatory effects of BKM120 on LC3-II levels, we checked the difference with or without CQ, which is a lysosomal protease inhibitor. In Figure 1C, the presence of CQ, which blocks autophagic degradation, further increased the levels of LC3-II compared to those in cells exposed to BKM120 alone, indicating that BKM120 indeed induces autophagic flux. Moreover, we detected significantly increased cells with punctuate pattern of YFP-LC3 when treated with BKM120 compared with those exposed to DMSO control (Figure 1D). The punctuate pattern indicates the formation of autophagosomes. Thus, our data first determined that BKM120 induces autophagy in lung cancer cells.

### Inhibition of autophagy enhances the effects of BKM120 on suppressing cell growth and induction of apoptosis

**Figure 2:**
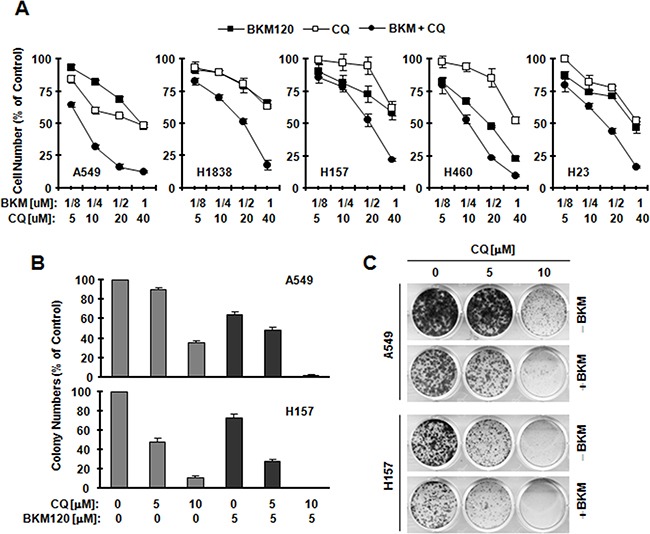
BKM120 combined with CQ synergistically inhibits the growth of human NSCLC cells **A.** The indicated cell lines were seeded in 96-well plates and treated the next day with different concentrations of CQ alone, BKM120 alone and their respective combinations as indicated. After 3 days, cell numbers were estimated using the SRB assay. CIs were calculated with CompuSyn software and labeled inside the graph. The data are means ± SDs of four replicate determinations. **B** and **C**, the indicated cell lines at a density of approximately 200 cells/well were seeded in12-well cell culture plates. On the second day, cells were treated with CQ, BKM120, and their combinations as indicated. The same treatments were repeated every 3 days. After 12 days, the plates were stained for the formation of cell colonies with crystal violet dye. Columns are means ± SD of triplicate measurements.

To analyze whether it is a killing or protective mechanism, we treated cells with CQ and then checked cell growth and apoptosis by BKM120 in human NSCLC cells, which are relative insensitive to BKM120 [9] and exhibit strong induction of LC3-II by BKM120 based on the results presented in Figure 1. In Figure 2A, the combination of BKM120 and CQ shows a better effect in inhibiting the growth in tested NSCLC cells lines than either agent alone. Further, treating cells with BKM120 and CQ shows a better suppressing effect in colony formation experiment (Figure 2B and 2C). All of above shows that blocking autophagy enhances BKM120's inhibitory effect.

**Figure 3:**
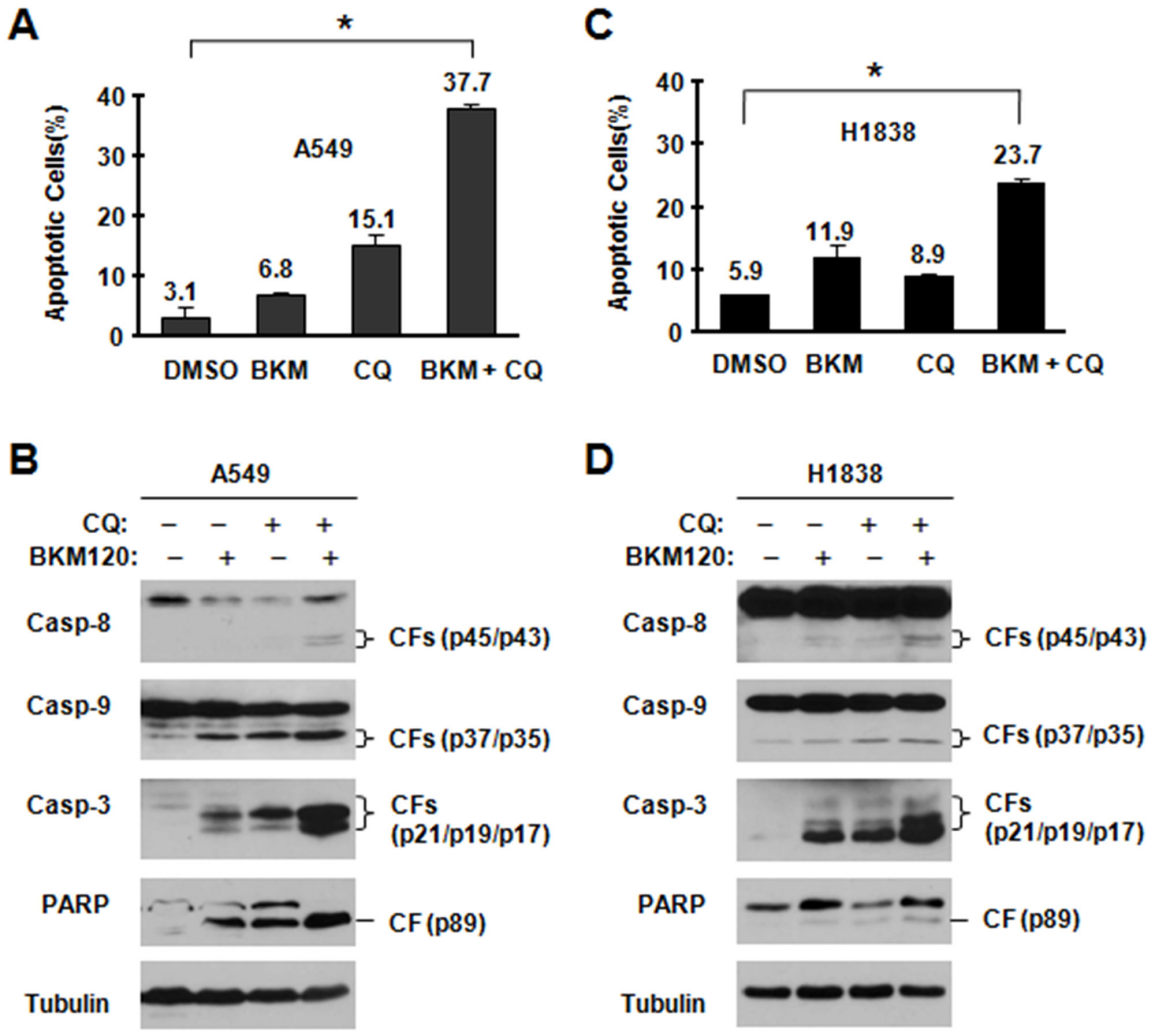
BKM120 and CQ combination enhances induction of apoptosis The indicated cell lines were treated with 2 μM BKM120, 40 μM CQ and their combination. After 48 h, the cells were harvested for detection of caspase and PARP cleavage with Western blot analysis **A, C.** and for measurement of apoptosis using Annexin V staining **B, D.** and Columns are means ± SDs of duplicate measurements. CF, cleaved form. * *P*<0.05.

Next, we analyzed apoptosis in tested cell lines. We treated cells with BKM120 and CQ and their combination respectively. As shown in Figure 3A and 3C, in tested cell lines (A549 and H1838), both BKM120 and CQ mildly induced apoptosis; however, compared with the single agent, the combination of these two agents showed significantly higher potency in inducing apoptosis. Protein PARP and cleaved caspase-3 were observed more in combination groups in western blotting assay (Figure 3B and 3D). Thus, the data shows that blocking autophagy enhances the ability of BKM120 to induce apoptosis.

### BKM120 increases p-ERK while inhibiting the PI3K/Akt axis in NSCLC cells

**Figure 4:**
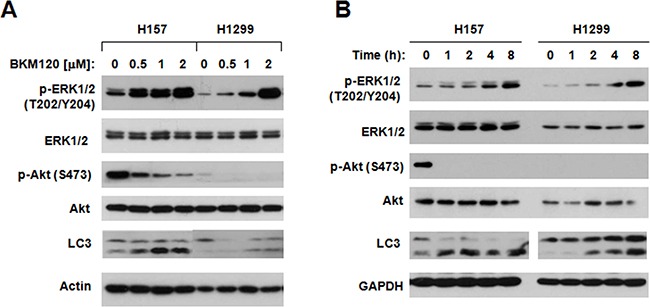
BKM120 increases p-ERK1/2 levels while inhibiting Akt phosphorylation The indicated cell lines were exposed to different concentrations of BKM120 as indicated for 8 h **A.** or with 2 μM BKM120 for the indicated times **B.** The cells were then harvested for preparation of whole-cell protein lysates and subsequent Western blot analysis to detect the given proteins.

There is a cross talk between PI3K/Akt and MEK/ERK signaling pathways. Some study reported that they can regulate each other's activity [14–21]. It has been shown that chemical MEK inhibitor may have a feedback effect to activate PI3K/Akt signaling in certain type cells [17, 22]. Thus, it is interesting whether blocking of the PI3K/Akt pathway with BKM120 induces activation of the MEK/ERK signaling in NSCLC cells. To this end, we treated H157 and H1299 with BKM120 at different concentrations for 8 h and then detected phosphorylation of ERK1/2, a well knows readout of the MEK/ERK signaling. BKM120 at concentrations of 0.5 to 2 μM, which decreased p-Akt levels, increased p-ERK1/2 levels in a concentration-dependent fashion in tested cell lines (Figure 4A). Increased levels of p-ERK1/2 were detected early at 1 h post BKM120 treatment and became even radical as the treatment times prolonged (Figure 4B). Thus it is clear that BKM120 upregulates the MEK/ERK signaling pathway while inhibiting the PI3K/Akt axis.

### The combination of BKM120 with a MEK inhibitor suppresses cell growth synergistically

**Figure 5:**
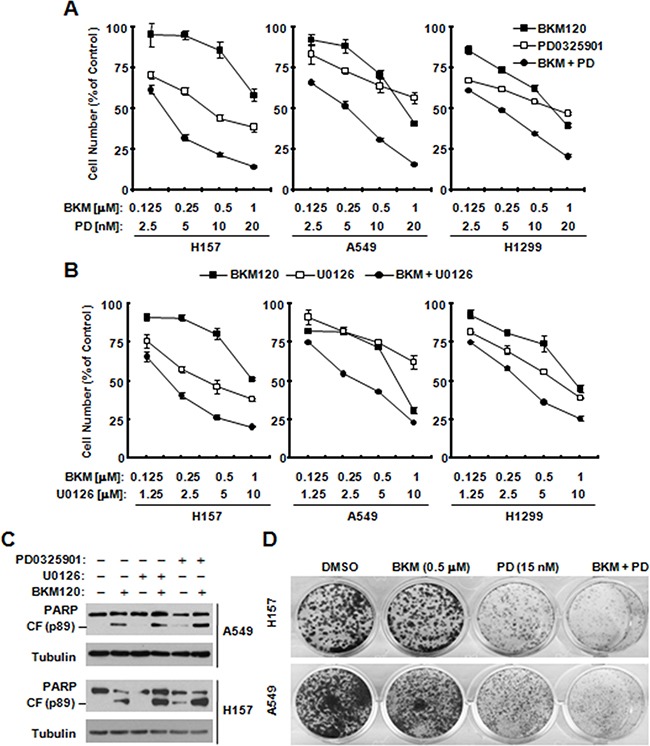
The combination of BKM120 with a MEK inhibitor synergistically inhibits the growth of human NSCLC cells (A, B and D) and enhances induction of apoptosis C *A and B*, The indicated cell lines were seeded in 96-well plates and treated the next day with different concentrations of PD0325901 (PD) alone, BKM120 alone and their respective combinations as indicated (*A*) or with different concentrations of U0126 alone, BKM120 alone and their respective combinations (*B*). After 3 days, cell numbers were estimated using the SRB assay. The data are means ± SDs of four replicate determinations. **C.** The indicated cell lines were treated with 2 μM BKM120 in the absence and presence of 20 nM PD0325901 or 20 μM U0126 for 24 h (H157) or 36 h (A549) and then harvested for preparation of whole-cell protein lysates and subsequent Western blot analysis to detect the given proteins. **D.** the indicated cells were treated with PD0325901, BKM120, and their combinations as indicated. The same treatments were repeated every 3 days. After 12 days, the plates were stained for the formation of cell colonies with crystal violet dye. The representative pictures of the colonies were taken as showed.

Given that BKM120 activates the MEK/ERK signaling as we demonstrate above, we then determined whether co-inhibition of PI3K and MEK strengthen inhibitory effects. The combination of BKM120 with either PD0325901 or U0126 exhibited more potent inhibitory effects than either one (Figure 5A and 5B). Moreover, the combination was also more potent than BKM120 alone (Figure 5C), indicating enhanced induction of apoptosis. Consistently, the colony formation assay also showed the similar result of synergistic inhibitory effects (Figure 5D). Hence, the data demonstrates clearly that the combination of these two pathway inhibitor synergistically suppressed lung cancer cell growth. The results also suggest that BKM120-induced MEK/ERK activation is a protective mechanism that attenuates BKM120's growth-inhibitory activity.

### MEK/ERK activation contributes BKM120-induced autophagy

**Figure 6:**
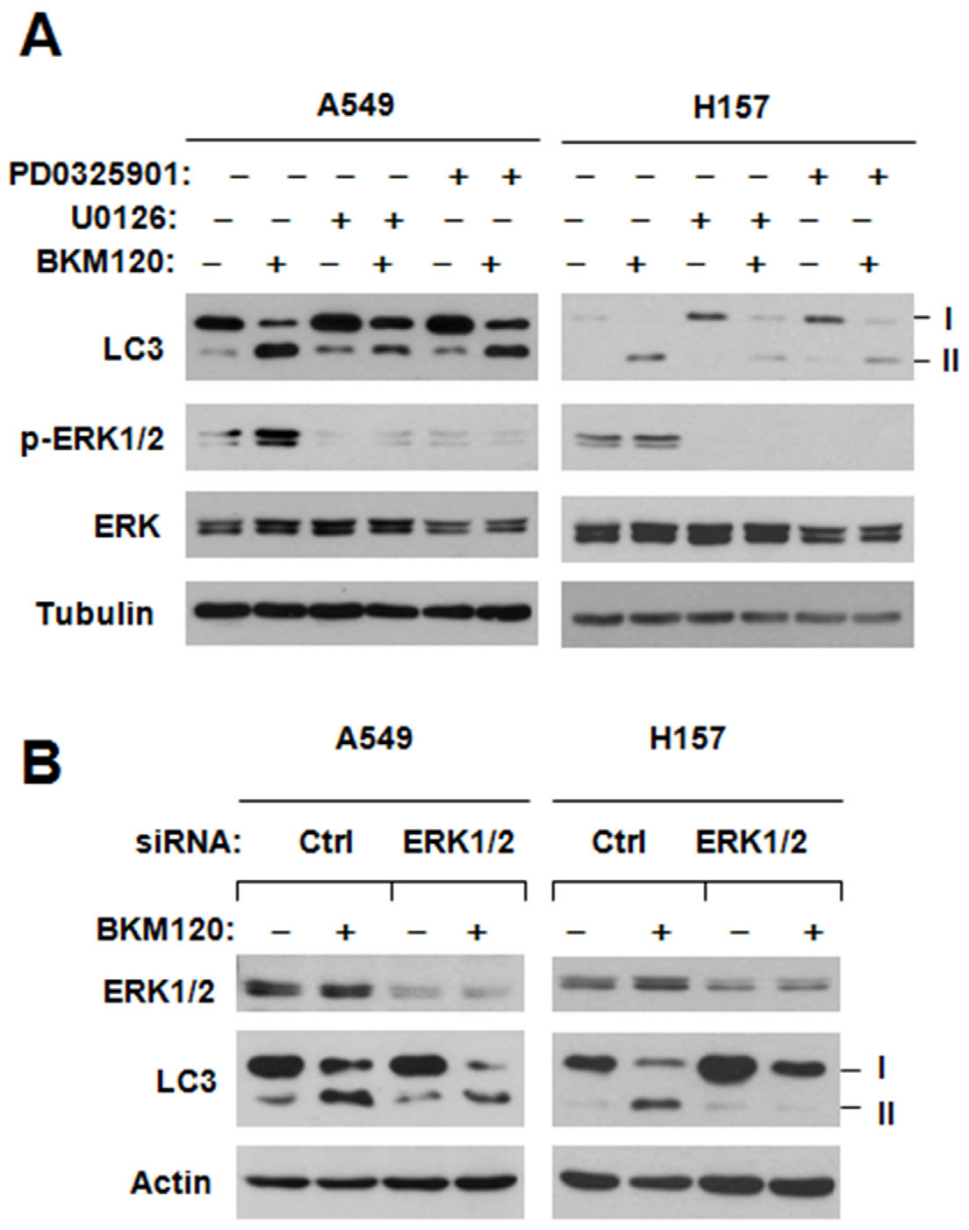
Chemical inhibition of MEK A. or siRNA-mediated ERK inhibition B. impairs the ability of BKM120 to elevate LC3-II levels *A,* The experimental conditions are same as described for Figure 5C. *B,* The indicated cell lines transfected with control (Ctrl) or ERK1/2 siRNA for 78 h were exposed to 2 μM BKM120 for additional 12 h. Then the cells were harvested for preparation of whole-cell protein lysates and subsequent Western blot analysis to detect the given proteins.

The MEK/ERK signaling is involved in positive regulation of autophagy [5]. Since both autophagy induction and activation of MEK/ERK signaling by BKM120 are protective events that attenuate BKM120's growth-inhibitory effects, we wondered whether there is a connection between MEK/ERK activation and autopahgy induction by BKM120. Thus, we first checked the LC3-II levels by BKM120 when combining with a MEK inhibitor or not. As shown in Figure 6A, the presence of either PD0325901 or U0126, at the tested concentrations that blocked ERK1/2 phosphorylation, clearly abolished BKM120's ability of increase LC3-II levels in both A549 and H157 cells. Moreover, we used ERK1/2 siRNA to silence ERK1/2 expression and then looked at its impact on BKM120-induced LC3-II elevation. In agreement with the data generated with MEK inhibitors, we detected much less amounts of LC3-II in ERK1/2 siRNA-transfected group than in control group (Figure 6B), indicating that inhibition of ERK1/2 attenuates the ability of BKM120 to increase LC3-II levels. These data together suggest that BKM120-induced autophagy involves activation of the MEK/ERK signaling.

### BKM120 synergizes with MEK/ERK inhibitor to inhibit the tumor growth in A549 xenografts

**Figure 7:**
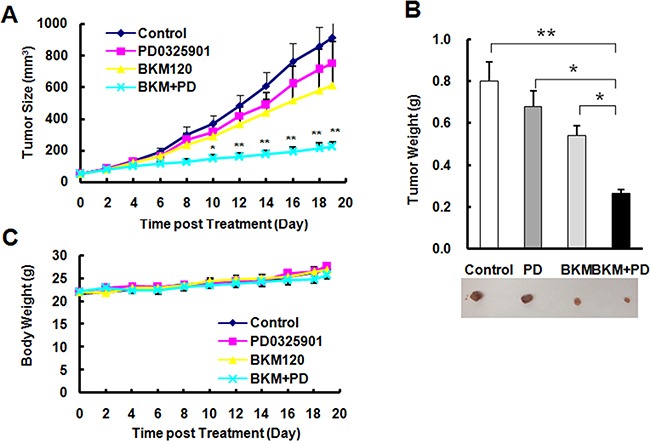
The combination of PD0325901 and BKM120 is significantly more effective than each single agent in suppressing the growth of NSCLC xenografts A and B without apparent toxicity in mice **C.** *A*, A549 xenografts were treated (once a day) with vehicle control, PD0325901, BMK120 and their combination (PD+BKM) starting on the same day after grouping. Tumor sizes (*A*) and mouse body weights (*C*) were measured as indicated. Each measurement is mean ± SEM (n = 6). After 19 days, the mice were sacrificed and the tumors were removed and weighed (B). *P < 0.05, **P < 0.01, compared with all other three treatment groups.

Based on the data in vitro, we further checked the inhibitory efficacy of the combination of BKM120 and PD0325901 in mouse xenografts. The combination group significantly suppresses the tumor growth (*P* < 0.01 compared with control group, *P* < 0.05 compared with PD0325901 orBKM120 group), whereas single agent doses only minimally suppressed tumor growth as measured by both tumor sizes (Figure 7A), and weights (Figure 7B). The data of body weight loss didn't show the significant difference (Figure 7C) in all groups during the whole experiment, suggesting that it is well tolerated. These in vivo data provide the same result as in vitro that thecombination of BKM120 and PD0325901 displays a synergistically inhibitory effect.

## DISCUSSION

The data has shown that BKM120 at concentrations ranges that effectively suppress the PI3K/Akt signaling potently induces autopagy, particularly in those cell lines that are relatively insensitive to BKM120, evidenced by increasing LC3-II and autophagosome-bound punctate pattern of YFP-LC3 (Figure 1). To the best of our knowledge, induction of autophagy by BKM120 in cancer cells has not been reported. In this project, autophagy by BKM120 clearly shows a protective effect based on the following findings: 1) stronger LC3-II was detected in cell lines that are less sensitive (e.g., H157, A549 and H1838) than in cell lines that are sensitive to BKM120 (e.g., H460 and H23); 2)when combines with CQ, BKM120 shows synergistic effects on suppressing cell growth (Figure 2A); 3) the combination exhibits enhanced suppressing effect in a colony formation assay (Figure 2B and 2C); and 4) BKM120-induced apoptosis is enhanced when combines with CQ (Figure 3). Given that CQ is a marketed anti-malarial drug [5], our findings hence warrant further evaluation of BKM120 and CQ combination as a therapeutic regimen against NSCLC and other types of cancers *in vivo* and in the clinic. This may be particularly useful for treatment of tumors that are relatively insensitive to BKM120 monotherapy.

It is well known that class I PI3K/Akt/mTORC1 signaling negatively regulates autophagy. Inhibition of this pathway with a class I PI3K inhibitor or mTOR inhibitor (e.g., rapamycin) accordingly induces autophagy [23]. Thus, the result on induction of autophagy by BKM120, a new pan-class I PI3K inhibitor, should be expected. On the other hand, we can assume that inhibition of the PI3K/Akt/mTORC1 signaling should be a reasonable mechanism accounting for BKM120-induced autophagy.

In addition to the negative regulation of autophagy by the class I PI3K/Akt/mTORC1 signaling [24–28], the MEK/ERK signaling is suggested to be involved in positive regulation of autophagy [29–32]. In the study, we could detect increased levels of p-ERK1/2 accompanied with reduced p-Akt in cells treated to BKM120 (Figure 4), suggesting that BKM120 activates the MEK/ERK signaling while inhibiting the PI3K/Akt signaling in the tested cell lines. Under the conditions that the MEK/ERK signaling or ERK1/2 was inhibited (e.g., with chemical MEK inhibitors or ERK1/2 siRNA), the ability of BKM120 to induce apoptosis was increased (Figure 5) and elevation of LC3-II levels was substantially impaired or attenuated (Figure 6). This enhanced growth-inhibitory effect was further validated in vivo using a A549 xenografts. The data suggested that the combination of PD0325901 and BKM120 was well tolerated in mice, but significantly suppressed tumor growth in mice in comparison with either agent alone, which only weakly inhibited tumor growth (Figure 7).

Thus, these data clearly indicate that BKM120-induced elevation of LC3-II or autopahgy is at least in part MEK/ERK-dependent. In agreement with what we generated with CQ, the presence of a MEK inhibitor, which blocked BKM120-induced ERK activation, substantially enhanced the effects not only on suppressing cell growth but also inducing apoptosis by BKM120 in a synergistic fashion. This further supports a role of MEK/ERK activation in mediating BKM120-induced autophagy. Hence we reasonably suggest that BKM120 is likely to induce autophagy by blockage of PI3K/Akt/mTOR signaling and activation of the MEK/ERK signaling. Our results also provide a strong scientific rationale for effective treatment of cancers such as NSCLC with BKM120 in combination with a MEK inhibitor.

To sum up, this study has revealed that the pan-class I PI3K inhibitor BKM120 induces autophagy, likely through increasing of the p-ERK and inhibition of the PI3K/Akt/mTOR axis. Blockage of the autopahgy either with a lysosomal protease inhibitor or MEK inhibitor will enhance BKM120's anticancer activity by augmenting its growth-inhibitory and apoptosis-inducing activity.

## MATERIALS AND METHODS

### Reagents

BKM120 was purchased from Selleck Chemicals (Houston, TX). BKM120 were dissolved in DMSO and stored at -80°C. We purchased MEK inhibitors, PD0325901 and U0126, from LC laboratories (Woburn, MA) and Selleck Chemicals (Houston, TX), respectively. Chloroquine (CQ), polyclonal and monoclonal actin antibodies were purchased from Sigma Chemical Co. (St. Louis, MO). Mouse monoclonal ERK1/2, p-ERK1/2 (Thr202/Tyr204), PARP, Akt, p-Akt (S473) antibodies were purchased from Cell Signaling Technology, Inc. (Danvers, MA). Mouse monoclonal caspase-3 antibody was purchased from Imgenex (San Diego, CA). Rabbit polyclonal LC3 antibody (NB100-2220) was purchased from Novus Biologicals, Inc. (Littleton, CO).

### Cell lines and cell culture

The lung cancer cell lines of H157, A549, H1299, H1838, H460 and H23 cell lines were purchased from the American Type Culture Collection(Manassas, VA). A549/YFP-LC3 stable lines were provided by Dr. Shi-Yong Sun (Emory University) [33]. These cell lineswere all maintained in RPMI 1640 medium supplemented with 10% fetal bovine serum at 37°C in a humidified atmosphere containing 5% CO_2_.

### Cell growth assay

Cells were seeded in 96-well plates and treated the next day. After 3 days, we discarded the medium, and fixed the adherent cells using 100 μl/well of 10% cold trichloroacetic acid for 60 min at cold room. We washed the plates 3 times with de-ionized water and air dried. 50 ul of 0.4% sulforhodamine B(SRB, Sigma, Louis, MO) were added for 10 min at ambient temperature. We further washed the plates 3 times with 1% acetic acid. Air dried and added 100 μl of Tris base (pH 10.5, 10 mM), and read OD at 492 nm.

### Detection of apoptosis

We analyzed apoptosis by flow cytometry using annexin V-PE apoptosis kit. We also detected cleavaged PARP, cleavaged caspase-9, cleavaged caspase-8, as well as caspase-3 by Western blot.

### Colony formation assay

Approximately 200 cells/well were seeded in 12-well plates and treated on the second day. Treatment medium was replaced every 3 days. After 12 days, we removed the medium and stained with crystal violet (0.1% in 20% methanol). The representative pictures of the colonies were taken using a digital camera and colonies were counted manually.

### Western blot analysis

Preparation of whole-cell protein lysates and Western blot analysis were described previously reference. Specific binding was detected using a chemiluminescence system (Millipore).

### Small interfering RNA (siRNA)-mediated gene silencing

ERK1/2 siRNA (Cell Signaling Technology, Inc. No. 6560) transfection was done using HiPerFect transfection reagent (Qiagen, Valencia, CA) following the manufacturer's instructions. Gene silencing effects were checked by Western blot.

### Lung cancer xenografts and treatments

Six week old male athymic (nu/nu) mice were housed under pathogen-free conditions in microisolator cages with laboratory chow and water. A549 cells at 8×10^6^ in serum-free medium were injected into the flank region of nude mice. When tumors reached a size of approximately 100 mm^3^, the mice were randomized into four groups (n = 6/group) according to tumor volumes and body weights for the following treatments: vehicle control, PD0325901 [(5 mg/kg/day, oral gavage (o.g)], BMK120 (7.5 mg/kg/day, o.g), and their combination. Tumor volumes were measured using caliper measurements once every two days and calculated with the formula V = π(length × width2)/6. After a consecutive 19-day treatment, the mice were sacrificed with CO2. The tumors were then removed and weighed.

### Statistical analysis

Every experiments repeated at least 3 times. The statistical significance of differences in tumor sizes or weights between two groups was analyzed with two-sided unpaired Student's *t* tests when the variances were equal or with Welch's corrected *t* test when the variances were not equal. *P* value < 0.05 was considered the threshold for statistical significance.
